# A study of laparoscopic extraperitoneal sigmoid colostomy after abdomino-perineal resection for rectal cancer

**DOI:** 10.1093/gastro/got036

**Published:** 2014-01-08

**Authors:** Jin Heiying, Du Yonghong, Wang Xiaofeng, Yao Hang, Wu Kunlan, Zhang Bei, Zhang Jinhao, Leng Qiang

**Affiliations:** National Center of Colorectal Surgery, The 3^rd^ Affiliated Hospital of Nanjing University of Traditional Chinese Medicine

**Keywords:** rectal cancer, abdomino-perineal resection (APR), laparoscopy, extraperitoneal ostomy, complication

## Abstract

**Objective:** To established a procedure for laparoscopic extraperitoneal ostomy after abdomino-perineal resection (APR) and study safety aspects and complications.

**Method:** From July 2011 to July 2012, 36 patients with low rectal cancer undergoing APR were included in the study and divided into extraperitoneal ostomy group (*n = *18) and intraperitoneal ostomy group (*n = *18). Short- and long-term complications were compared between the two groups. All patients were followed up and the median duration was 17 months (range: 12–24).

**Results:** The rates of short-term complication related to colostomies were comparable between the two groups, except the rate for stoma edema was higher in the extraperitoneal group (33.3% vs 0%; *P = *0.008). In the intraperitoneal ostomy group, two patients developed stoma prolapse, one had stoma stenosis, and two had parastomal hernia. In contrast, no long-term complications related to colostomies occurred in the extraperitoneal ostomy group. The rate of long-term complication was lower in the extraperitoneal ostomy group (0% vs 22.2%; *P = *0.036).

**Conclusion:** The laparoscopic extraperitoneal ostomy is a relatively simple and safe procedure, with fewer long-term complications related to colostomy. However the follow-up period was not too long and needs to be extended.

## INTRODUCTION

Sigmoid colostomy created through the extraperitoneal route has been reported to produce a reduced risk of associated parastomal hernia and stomal prolapse [1]. A meta-analysis of 1071 cases found that extraperitoneal sigmoid colostomy prevented parastomal hernia without increasing the risk of other post-operative complications such as stoma ischemia, obstruction and prolapse [2]. Laparoscopy is being widely used for the treatment of rectal cancer, but laparoscopic construction of an extraperitoneal colostomy is technically difficult. Also, concerns persist about complications that might occur with this approach, namely stoma ischemia and necrosis and the development of intestinal hernias due to insufficiency of peritoneum. Therefore, the intraperitoneal route for laparoscopic construction of sigmoid colostomies is usually preferred, even though it is known to carry a risk of stomal prolapse and parastomal hernia. Hauters *et al.* used an intraperitoneal onlay mesh reinforcement at the time of stoma formation to prevent parastomal hernia [3]. Indeed, Hamada *et al.* reported a high incidence of parastomal hernia by CT examination of intraperitoneal colostomies, and accordingly recommended that extraperitoneal colostomy be the preferred procedure [4]. Akamoto *et al.* designed a special hook to facilitate stoma formation [5]. Leroy *et al.* also recommended laparoscopic extraperitoneal colostomy to prevent parastoma hernia [6].

In this report, we set out the results of a randomized, controlled study of extraperitoneal and intraperitoneal sigmoid colostomy. Complications related to colostomies were of special interest.

## PATIENTS AND METHODS

### Patients

A single-institute, randomized and controlled trial was designed. Admission criteria were patients with distal rectal cancer undergoing abdomino-perineal resection (APR) at the National Center of Colorectal Surgery, the 3^rd^ Affiliated Hospital of Nanjing University of Traditional Chinese Medicine, between July 2011 and July 2012. Patients with synchronous metastases (M1) were excluded from the study. Patients were randomly assigned into two groups by the random table method: extraperitoneal or intraperitoneal ostomy. The study was approved by the hospital's ethics committee, and informed consent was obtained from each participating patient.

### Methods of colostomy construction

**Figure 1.**
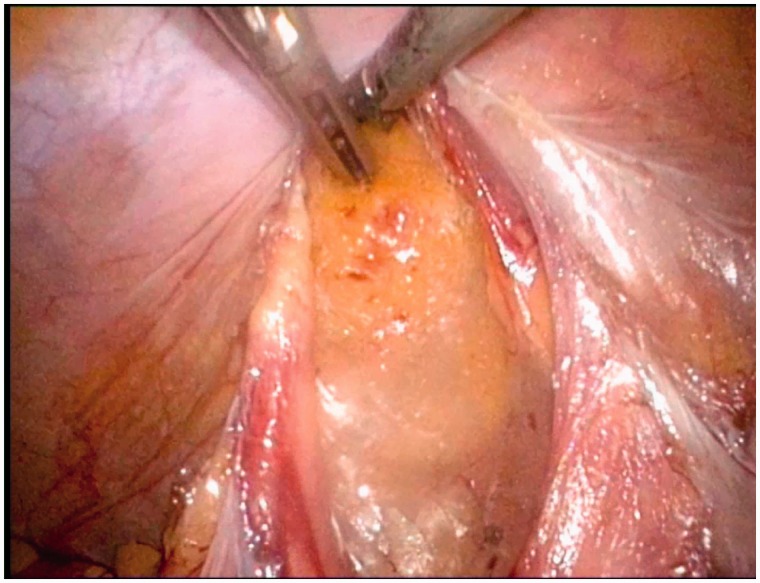
A 3–4 cm cavity was made in the left side of peritoneum as an internal opening of the extraperitoneal tunnel.

**Figure 2.**
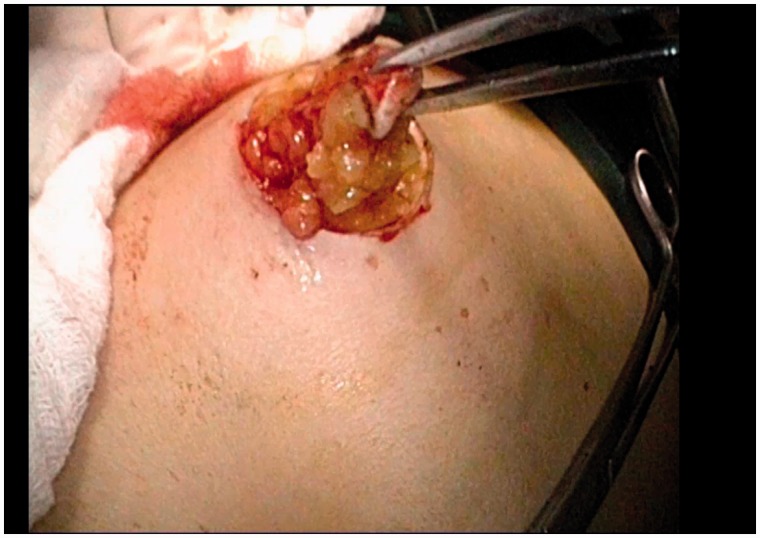
A circular incision of 2 cm diameter was made at the pre-planned position of the stoma.

**Figure 3.**
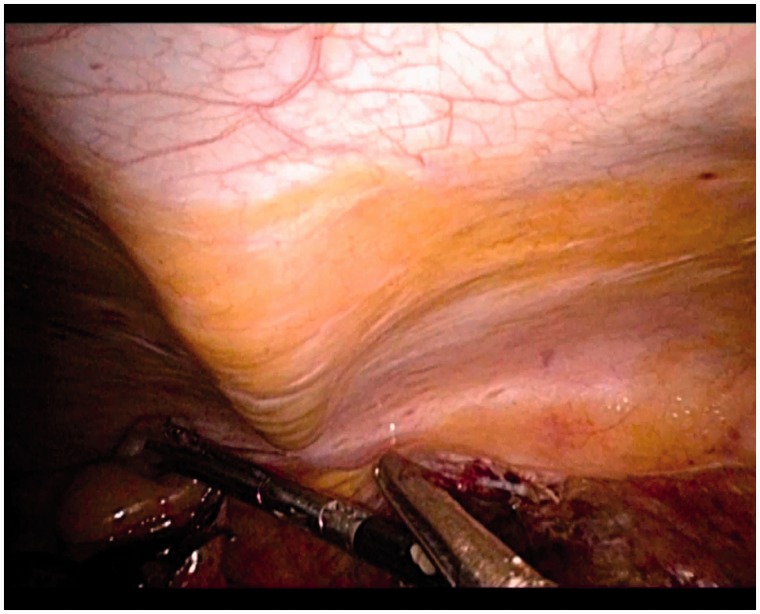
The peritoneum was blunt-separated by use of Kocher forceps to the internal opening of the extraperitoneal tunnel and the diameter of the tunnel was dilated to 2–3 finger widths.

**Figure 4.**
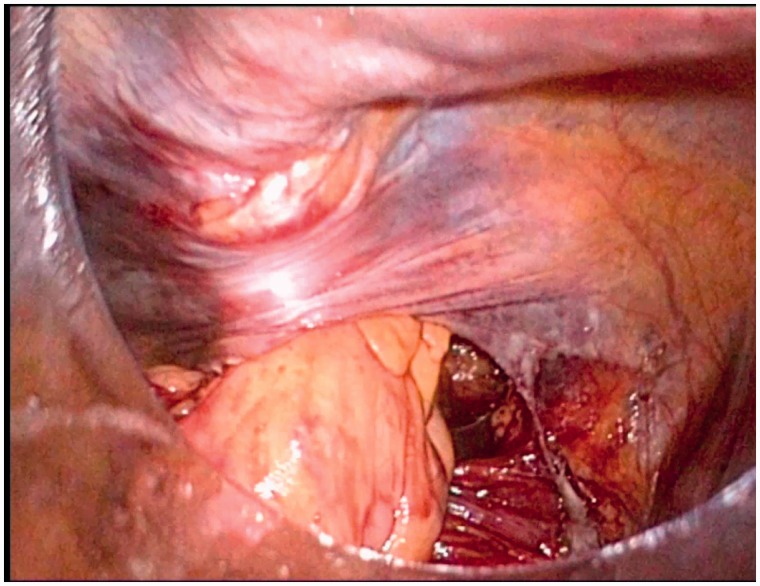
The proximal sigmoid colon was pulled out of the tunnel.

**Figure 5.**
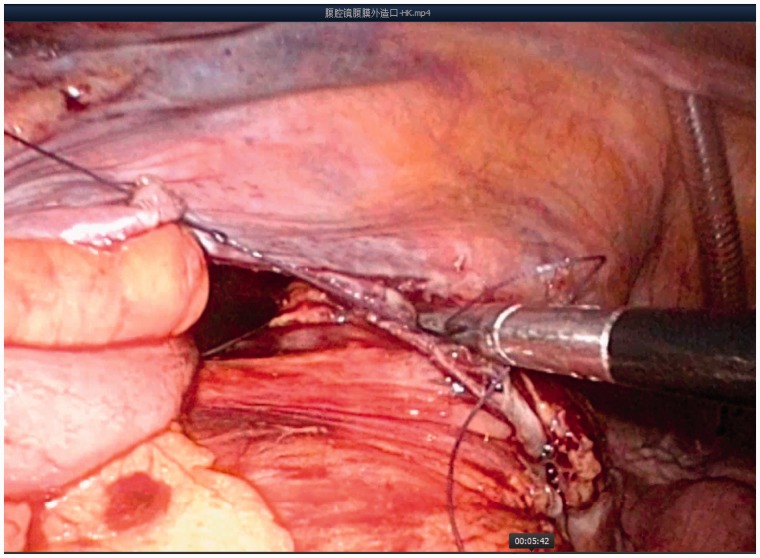
Sutured to cavity between the peritoneum and colon with one or two stitches.

**Figure 6.**
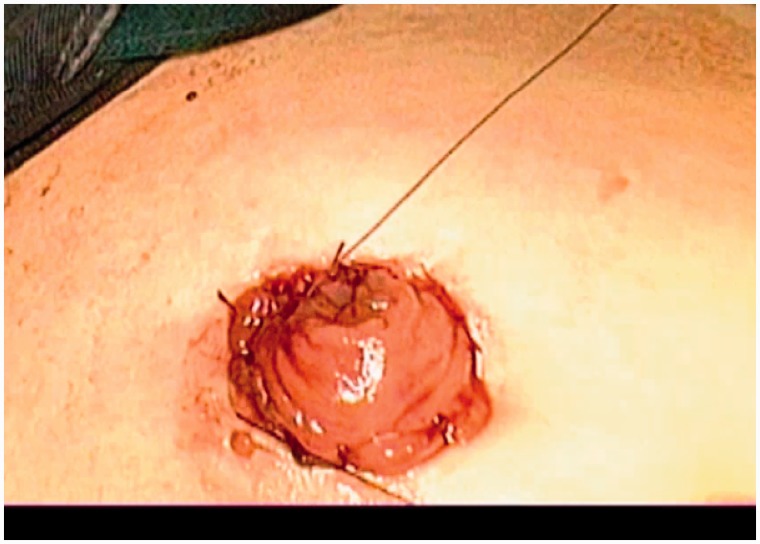
The intestinal wall and skin were stood out manually before the end of the operation.

Intraperitoneal colostomies were constructed by the conventional method [5]. Extraperitoneal colostomies were constructed as follows: the sigmoid colon was transected from the middle after the rectum was completely mobilized. The proximal sigmoid colon was mobilized in order that it could reach the abdominal wall without tension. A 3–4 cm cavity was made in the left side of the peritoneum as an internal opening of the extraperitoneal tunnel and marked with a no-damage clamp (Figure 1). A circular incision of 2 cm diameter was made at the pre-planned position of the stoma (Figure 2). The skin and subcutaneous tissues were removed and the anterior rectus sheath was opened with a cross incision. The rectus abdominis was separated, and 0.5 cm of muscle was cut off (the separation and removal is not necessary if the rectus abdominis is not very strong). The peritoneum was blunt-separated by use of Kocher forceps to the internal opening of the extraperitoneal tunnel and the diameter of the tunnel was dilated to two to three finger widths (Figure 3). The proximal sigmoid colon was pulled out of the tunnel (Figure 4) and sutured to the cavity between the peritoneum and colon with one or two stitches (Figure 5). The gap between the rectus sheath and the intestinal wall was closed with sutures. Finally, the intestinal wall and skin were sutured manually before the end of the operation (Figure 6).

### Follow-up examinations and statistical analysis

Status of the stomas was closely monitored during hospital stay and was periodically reviewed at follow-up outpatient examinations. Short-term complications were defined as complications that occurred within 4 weeks, such as hemorrhage, ischemia, dermatitis and edema. Long-term complications were defined as complications that occurred after 4 weeks, such as prolapse, narrow stoma, retraction, and parastomal hernia. The count data were presented as a ratio and analysed by Fisher's exact test. The measurement data were expressed as median ± standard deviation and analysed with the Student's *t-*test.

## RESULTS

From July 2011 to July 2012, 36 patients undergoing APR were included in this study and randomly divided into intraperitoneal ostomy group (*n = *18) and extraperitoneal ostomy group (*n = *18). Three of the 36 patients had received pre-operative neoadjuvant chemoradiotherapy. One patient in each group underwent reconstruction of the stoma because of insufficient blood supply to the colostomy. Patient and tumor characteristics are described in Table 1.

**Table 1.** Follow-up information and complications of laparoscopic sigmoid colostomy constructed through the extraperitoneal or intraperitoneal routes

	Intraperitoneal (*n = *18)	Extraperitoneal (*n = *18)	*P*-value
Age (years)	59.7 ± 14.4	61.4 ± 11.4	0.388
Gender, *n* (%)			0.508
Male	7 (38.9)	9 (50.0)	
Female	11 (61.1)	9 (50.0)	
BMI (kg/m^2^)	22.8 ± 2.0	23.1 ± 2.7	0.095
Stage of tumor			0.453
Stage I	4 (22.2)	4 (22.2)	
Stage II	2 (11.1)	5 (27.8)	
Stage III	12 (66.7)	9 (50.0)	
Operative time (min)	25.3 ± 8.5	14.7 ± 6.4	0.062
Short-term complications, *n* (%)	8 (44.4)^a^	5 (27.8)	0.148
Hemorrhage	1 (5.6)	1 (5.6)	1.000
Ischemia	1 (5.6)	1 (5.6)	1.000
Retraction	0	0	1.000
Dermatitis	2 (11.1)	3 (16.7)	0.791
Edema	6 (33.3)	0	0.008
Long-term complications, *n* (%)	0	4 (22.2)	0.036
Prolapse	0	1 (5.6)	0.317
Narrow stoma	0	1 (5.6)	0.317
Retraction	0	0	–
Hernia	0	2 (11.1)	0.151

^a^Two kinds of short-term complication occurred in two patients.

The mean operative time was 25.3 min in the extraperitoneal group, which was higher than that in the intraperitoneal group (14.7 min), but the difference did not reach statistical significance (*P = *0.062). All the patients were followed for 12–24 months (median: 17) after operation. The rates of short-term complication related to colostomies were comparable between the two groups (44.4% vs 27.8%; *P = *0.148) except for stoma edema was higher in extraperitoneal ostomy group (33.3% vs 0%, *P = *0.008). The rate of long-term complication related to colostomies was lower in the extraperitoneal ostomy group (0% vs 22.2%; *P = *0.036). In the intraperitoneal ostomy group, two patients developed stoma prolapse; one, stoma stenosis and two, parastoma hernia, whereas no long-term complication occurred in the extraperitoneal ostomy group (Table 1).

## DISCUSSION

A sphincter-preserving operation is widely used for the treatment of distal rectal cancer, but 10–20% of patients still prefer APR and permanent colostomy. However, post-operative complications of this procedure can adversely affect quality of life [8, 9]. Results of various studies have shown that extraperitoneal sigmoidostomy is associated with a low incidence of complications, mainly in the form of parastomal hernia and stomal prolapse. Surgeons have thus increasingly come to prefer this procedure [2, 10]. However, laparoscopic construction of a sigmoid colostomy is difficult. Extraperitoneal sigmoid ostomy is especially challenging because of difficulties encountered in closing the lateral peritoneum and pelvic floor. Also it has been questioned as to whether extraperitoneal colostomy will be therapeutically effective or have excessive surgical complications. Intraperitoneal colostomy was therefore adapted to the laparoscopic operation.

In the present study, we evaluated intraperitoneal and extraperitoneal colostomies constructed via laparoscopy. The extraperitoneal operation was similar to that of conventional open surgery but with an easier operative technique. The average operation time was 25 minutes, close to those reported by others [3, 4], and was only 10 min longer than that of intraperitoneal colostomy, without adversely affecting prognosis. The incidence of short-term complications—such as stomal ischemia or hemorrhage—was similar between extraperitoneal- and intraperitoneal colostomy patients. Two patients in each group required a second operation within 5–6 days of the initial one because of stomal retraction due to thrombosis, with subsequent ischemia and necrosis. In obese patients, extracting the proximal sigmoid can be difficult. We managed this problem by expanding the peritoneal tunnel to a diameter of at least two finger widths, so that the mesentery could be removed along with the resected sigmoid colon, as long as there was sufficient blood supply.

Stomal edema occurred in one-third of patients who had extraperitoneal colostomy but did not occur in any of those who had intraperitoneal colostomy. This complication may be ascribed to poor intestinal blood circulation resulting from compression by the tunnel. In all patients, the stomal edema resolved, without specific treatment, within two weeks of the operation.

The main rationale for using the extraperitoneal method of stomal construction is the possible decrease in associated long-term complications, such as parastomal hernia, stomal prolapse, and stomal retraction [11, 12]. Our results justify the use of this approach, since the post-operative complication rate was lower in patients with an extraperitoneal stoma than in intraperitoneal. Indeed, there were no long-term complications in the group of extraperitoneal colostomy patients whereas, in the intraperitoneal colostomy group, there was one instance of parastomal hernia, two of stomal prolapse, and one of stomal stenosis. We suspect that the stomal stenosis resulted from drainage of a parastomal abscess and subsequent scar formation. Although the differences in complication rates between the two patient groups did not reach statistical significance, this may have reflected the small number of patients involved. It also is possible that more complications would have become evident had the follow-up period been longer.

In conclusion, the laparoscopic extraperitoneal ostomy is an easy and safe procedure. It did not increase complications following the operation. The long-term complications were lower in the extraperitoneal ostomy group. However, the follow-up period was short and longer follow up is needed.

**Conflict of interest:** none declared.

## References

[1] Carne PW, Robertson GM, Frizelle FA (2003). Parastomal hernia. Br J Surg.

[2] Lian L, Wu XR, He XS (2012). Extraperitoneal vs intraperitoneal route for permanent colostomy: a meta-analysis of 1,071 patients. Int J Colorectal Dis.

[3] Hauters P, Cardin JL, Lepere M (2012). Prevention of parastomal hernia by intraperitoneal onlay mesh reinforcement at the time of stoma formation. Hernia.

[4] Hamada M, Ozaki K, Muraoka G (2012). Permanent end-sigmoid colostomy through the extraperitoneal route prevents parastomal hernia after laparoscopic abdomino-perineal resection. Dis Colon Rectum.

[5] Akamoto S, Noge S, Uemura J (2013). Extraperitoneal colostomy in laparoscopic abdomino-perineal resection using a laparoscopic retractor. Surg Today.

[6] Leroy J, Diana M, Callari C (2012). Laparoscopic extraperitoneal colostomy in elective abdomino-perineal resection for cancer: a single surgeon experience. Colorectal Dis.

[7] Scheidbach H, Schneider C, Konradt J (2002). Laparoscopic abdomino-perineal resection and anterior resection with curative intent for carcinoma of the rectum. Surg Endosc.

[8] Kasparek MS, Hassan I, Cima RR (2011). Quality of life after coloanal anastomosis and abdomino-perineal resection for distal rectal cancers: sphincter preservation vs quality of life. Colorectal Dis.

[9] Kim JS, Hur H, Kim NK (2009). Oncologic outcomes after radical surgery following pre-operative chemoradiotherapy for locally advanced lower rectal cancer: abdomino-perineal resection versus sphincter-preserving procedure. Ann Surg Oncol.

[10] Dong LR, Zhu YM, Xu Q (2012). Clinical evaluation of extraperitoneal colostomy without damaging the muscle layer of the abdominal wall. *J Int Med Res*,.

[11] Ahmad NZ, Racheva G, Elmusharaf H (2013). A systematic review and meta-analysis of randomized and non-randomized studies comparing laparoscopic and open abdomino-perineal resection for rectal cancer. Colorectal Dis.

[12] Simorov A, Reynoso JF, Dolghi O (2011). Comparison of perioperative outcomes in patients undergoing laparoscopic versus open abdomino-perineal resection. Am J Surg.

